# Adult Prostate Sarcoma: Demographics, Treatment Patterns, and Survival

**DOI:** 10.1245/s10434-024-16258-w

**Published:** 2024-09-23

**Authors:** Carolin Siech, Mario de Angelis, Francesco Di Bello, Natali Rodriguez Peñaranda, Jordan A. Goyal, Zhe Tian, Fred Saad, Shahrokh F. Shariat, Stefano Puliatti, Nicola Longo, Alberto Briganti, Séverine Banek, Philipp Mandel, Luis A. Kluth, Felix K. H. Chun, Pierre I. Karakiewicz

**Affiliations:** 1 Cancer Prognostics and Health Outcomes Unit, Division of Urology, University of Montréal Health Center, Montréal, QC Canada; 2 Goethe University Frankfurt, University Hospital, Department of Urology, Frankfurt am Main, Germany; 3 Division of Experimental Oncology/Unit of Urology, URI, IRCCS Ospedale San Raffaele, Milan, Italy; 4 Vita-Salute San Raffaele University, Milan, Italy; 5 Department of Neuroscience, Science of Reproduction and Odontostomatology, University of Naples Federico II, Naples, Italy; 6 Department of Urology, AOU di Modena, University of Modena and Reggio Emilia, Modena, Italy; 7 Department of Urology, Comprehensive Cancer Center, Medical University of Vienna, Vienna, Austria; 8 Department of Urology, Weill Cornell Medical College, New York, NY USA; 9 Department of Urology, University of Texas Southwestern Medical Center, Dallas, TX USA; 10 Hourani Center for Applied Scientific Research, Al-Ahliyya Amman University, Amman, Jordan

**Keywords:** Cancer-specific mortality, Prostate cancer, Mesenchymal tumor, Variant histology, SEER

## Abstract

**Background:**

This study aimed to examine clinicopathologic characteristics, treatment patterns, and survival rates in a contemporary population-based cohort of adult prostate sarcoma patients.

**Methods:**

In the Surveillance, Epidemiology, and End Results database (2004–2020), adult patients with prostate sarcoma were identified. Descriptive statistics, Kaplan–Meier analyses, smoothed cumulative incidence plots, and Cox regression models were used.

**Results:**

Of 125 patients, 45 (36%) harbored leiomyosarcoma, 17 (14%) had rhabdomyosarcoma, 15 (12%) had stromal sarcoma, 17 (14%) had sarcoma not otherwise specified (NOS), and 31 (25%) had other sarcoma subtypes. Metastatic stage was most common in the rhabdomyosarcoma patients (44%) and least common in the leiomyosarcoma (21%) and stromal sarcoma (20%) patients. Most of the rhabdomyosarcoma patients received the combination of systemic and radiation therapy with (24%) or without radical surgery (35%), whereas most of the leiomyosarcoma and stromal sarcoma patients underwent radical surgery with (22 and 13%) or without (22 and 47%) radiation. In the overall population, the median overall survival was 27 months. The 5-years overall versus cancer-specific versus other-cause mortality rates were respectively 71 versus 58 versus 13%. In the multivariable Cox regression models, the highest overall mortality was exhibited by the patients with metastatic disease (hazard ratio [HR] 2.87; 95% confidence interval [CI] 1.55–5.31; *p* < 0.001) or unknown disease stage (HR 2.94; 95% CI 2.20–7.21; *p* = 0.019). Conversely, of all the histologic subtypes, only stromal sarcoma distinguished itself by lower overall mortality (HR 0.41; 95% CI 0.18–0.96; *p* = 0.039).

**Conclusions:**

Four major histologic subtypes were identified. Among most adult sarcoma patients, treatment patterns vary according to histology, from multimodal therapy to radical prostatectomy alone. These treatment differences reflect equally important heterogeneity in survival patterns.

Non-epithelial malignancies represent rare prostate cancer subtypes.^1–3^ The majority of these arise from cells of mesenchymal origin, such as fibromuscular stroma and smooth muscle cells, and are therefore referred to as prostate sarcoma.^1^ Literature addressing adult prostate sarcoma patients is scarce.^4–12^ Most of the reports describe single-institutional series that rely on small samples and historic patient cohorts.^4–9,12^ Several analyses also have included pediatric patient subgroups.^4–7,11^ Furthermore, to the best of our knowledge, no contemporary analysis has addressed disease stage at presentation and treatment patterns in adult prostate sarcoma patients according to histologic subtypes.

We addressed these knowledge gaps. Specifically, we postulated that stage at presentation, treatment patterns, and overall survival (OS) differ according to histologic prostate sarcoma subtypes. Moreover, we also hypothesized that cancer-specific mortality (CSM) rates are higher than other-cause mortality (OCM) rates in the overall population as well as in stage-specific and histologic subtype-specific analyses. To test these hypotheses, we relied on a contemporary population-based cohort of adult patients with prostate sarcoma in the Surveillance, Epidemiology, and End Results (SEER 2004–2020) database.

## Materials and Methods

### Data Source and Study Population

The SEER database (2004–2020; https://seer.cancer.gov/data/) provides cancer statistics covering approximately 47.9% of the U.S. population.^13^ We focused on patients with histologically confirmed prostate sarcoma (International Classification of Diseases [ICD-10] site code C61). Specifically, we identified the following prostate sarcoma subtypes: leiomyosarcoma (International Classification of Disease for Oncology 3rd-edition [ICD-O-3] codes 8890/3), rhabdomyosarcoma (ICD-O-3 codes 8900/3, 8902/3, 8910/3, 8912/3, and 8920/3), sarcoma not otherwise specified (NOS; ICD-O-3 code 8800/3), stromal sarcoma (ICD-O-3 codes 8810/3, 8935/3, and 9020/3), and other sarcoma (ICD-O-3 codes 8801/3, 8802/3, 8805/3, 8806/3, 8815/3, 8830/3, 8936/3, 9040/3, 9041/3, 9120/3, 9260/3, 9364/3, and 9561/3).

The study included only adult patients (age ≥ 18 years) with primary diagnosis, known vital status, or cause of death. Autopsy or death certificate-only cases were excluded. The study was conducted in accordance with the principles set in the Helsinki Declaration. Because the study was based on the anonymously coded design of the SEER database, study-specific Institutional Review Board ethics approval was not required.

### Variables and Outcomes of Interest

The variables of interest were histologic subtypes, disease stage at presentation, and treatment patterns. Radical surgery was defined as pelvic exenteration (surgery-site code 70) or radical prostatectomy (surgery-site code 50). Radiation therapy included brachytherapy and external beam radiotherapy. The primary study end point was overall mortality (OM), defined as all deaths regardless of cause of death. The secondary study end points were CSM, defined as all cancer-related deaths, and OCM, defined as all deaths due to any cause except mortality from prostate cancer.

### Statistical Analyses

Four analytical steps were completed. First, demographics and treatment patterns were tabulated. For continuously coded variables, medians and interquartile ranges (IQR) were reported. For categorical variables, frequencies and proportions were recorded. Second, Kaplan-Meier survival analyses addressed OS first in the overall population of prostate sarcoma patients, then after stratification according to stage at presentation (localized versus locally advanced versus metastatic) and histologic subtypes (leiomyosarcoma versus rhabdomyosarcoma versus stromal sarcoma versus sarcoma NOS versus other sarcoma). Subsequently, smoothed cumulative incidence plots distinguished between CSM and OCM. Finally, uni- and multivariable Cox regression models were fitted to test for OM differences according to histologic subtype and disease stage at presentation.

All statistical tests were two sided, with a level of significance set at a *p* value lower than 0.05. The study used R software environment for statistical computing and graphics (R version 4.3.2; R Foundation for Statistical Computing, Vienna, Austria).^14^

## Results

### Patient and Tumor Characteristics of Prostate Sarcoma Patients

**Fig. 1:**
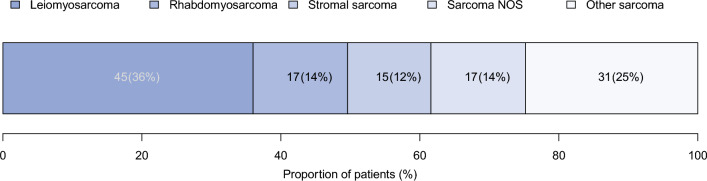
Distribution of histologic subtypes of adult patients with newly diagnosed sarcoma of the prostate in the Surveillance, Epidemiology, and End Results database (2004–2020). NOS, not otherwise specified

**Fig. 2:**
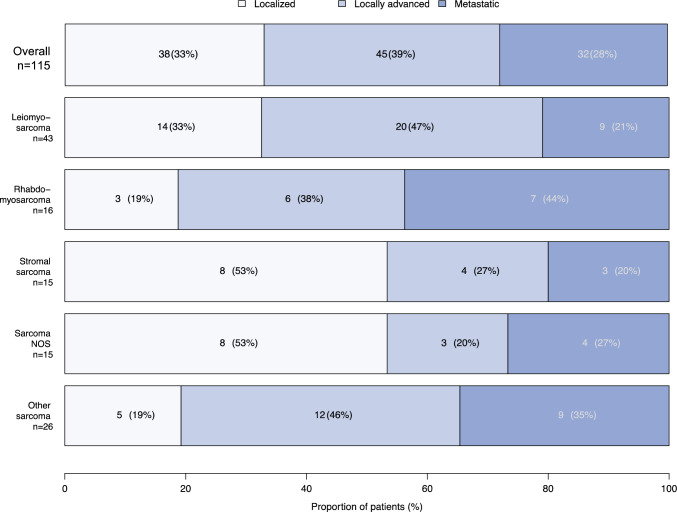
Stage distribution of adult patients with newly diagnosed sarcoma of the prostate according to histologic subtype in the Surveillance, Epidemiology, and End Results database (2004–2020). Due to unknown cancer stage at presentation, 10 of 125 patients were excluded from analyses. NOS, not otherwise specified

In the SEER database (2004–2020), we identified 125 patients with prostate sarcoma. The median age at diagnosis was 63 years (IQR, 49–73 years; Table 1). Of these patients, 45 (36%) harbored leiomyosarcoma, 17 (14%) had rhabdomyosarcoma, 15 (12%) had stromal sarcoma, and 17 (14%) had sarcoma NOS (Fig. 1). The remaining 31 (25%) patients were summarized as other histologic subtypes. Specifically, we identified 6 (5%) patients with spindle cell sarcoma, 4 (3%) with giant cell sarcoma, 4 (3%) with undifferentiated sarcoma, 4 (3%) with hemangiosarcoma, 3 (2%) with malignant solitary fibrous tumor, 2 (2%) with gastrointestinal stromal sarcoma, 2 (2%) with synovial sarcoma, 2 (2%) with Ewing sarcoma, 1 (1%) with desmoplastic small round cell tumor, 1 (1%) with malignant fibrous histiocytoma, 1 (1%) with peripheral neuroectodermal tumor, and 1 (1%) with malignant peripheral nerve sheath tumor with rhabdomyoblastic differentiation of the prostate. Metastatic stage was most prevalent in rhabdomyosarcoma (44%), followed by other sarcoma (35%), sarcoma NOS (27%), leiomyosarcoma (21%), and stromal sarcoma patients (20%; Fig. 2).

**Table 1:** Patient and tumor characteristics of adult patients with sarcoma of the prostate in the Surveillance, Epidemiology, and End Results database (2004–2020)

Characteristic		Overall (*n* = 125) *n* (%)
Age (years)^a^	Median (IQR)	63 (49–73)
Race/ethnicity	Caucasians	84 (67)
	Hispanics	15 (12)
	African Americans	8 (6)
	Others	18 (14)
Histologic subtype	Leiomyosarcoma	45 (36)
	Rhabdomyosarcoma	17 (14)
	Stromal sarcoma	15 (12)
	Sarcoma NOS	17 (14)
	Other sarcoma	31 (25)
Stage	Localized	38 (30)
	Locally advanced	45 (36)
	Metastatic	32 (26)
	Unknown	10 (8)

IQR, interquartile range; NOS, not otherwise specified

### Treatment Patterns of Prostate Sarcoma Patients

Of 45 leiomyosarcoma patients, 30 (67%) were treated with radical surgery, 19 (42%) received radiation therapy, and 13 (29%) received systemic therapy (Table 2). Of 17 rhabdomyosarcoma patients, 13 (76%) received systemic therapy, 12 (71%) received radiation therapy, and 8 (47%) were treated with radical surgery. Of 15 stromal sarcoma patients, 9 (60%) were treated with radical surgery, 3 (20%) received radiation therapy, and 1 (7%) received systemic therapy. Of 17 sarcoma NOS patients, 8 (47%) were treated with radical surgery, 5 (29%) received systemic therapy, and 3 (18%) received radiation therapy. Finally, of 31 other sarcoma patients, 16 (52%) received radiation therapy, 13 (42%) were treated with radical surgery, and 11 (35%) received systemic therapy.

**Table 2:** Treatment patterns of adult patients with newly diagnosed sarcoma of the prostate in the Surveillance, Epidemiology, and End Results database (2004–2020)

Definitive treatment	Overall (*n* = 125) *n* (%)	Leiomyo-sarcoma (*n* = 45, 36%) *n* (%)	Rhabdomyo-sarcoma (*n* = 17, 14%) *n* (%)	Stromal sarcoma (*n* = 15, 12%) *n* (%)	Sarcoma NOS (*n* = 17, 14%) *n* (%)	Other sarcoma (*n* = 31, 25%) *n* (%)
Treatment combinations						
No treatment	27 (22)	9 (20)	1 (6)	4 (27)	5 (29)	8 (26)
Systemic therapy alone	9 (7)	2 (4)	1 (6)	1 (7)	3 (18)	2 (7)
Radiation alone	11 (9)	3 (7)	1 (6)	1 (7)	1 (6)	5 (16)
Radical surgery alone	27 (22)	10 (22)	1 6)	7 (47)	5 (29)	4 (13)
Systemic therapy and radiation	10 (8)	1 (2)	6 (35)	0 (0)	0 (0)	3 (10)
Systemic therapy and radical surgery	9 (7)	5 (11)	2 (12)	0 (0)	1 (6)	1 (3)
Radiation and radical surgery	17 (14)	10 (22)	1 (6)	2 (13)	1 (6)	3 (10)
Systemic therapy and radiation and radical surgery	15 (12)	5 (11)	4 (24)	0 (0)	1 (6)	5 (16)
Systemic therapy	43 (34)	13 (29)	13 (76)	1 (7)	5 (29)	11 (35)
Radiation therapy	53 (42)	19 (42)	12 (71)	3 (20)	3 (18)	16 (52)
Radical surgery	68 (54)	30 (67)	8 (47)	9 (60)	8 (47)	13 (42)
Pelvic exenteration	31 (46)	15 (50)	6 (75)	1 (11)	1 (13)	8 (62)
Radical prostatectomy	37 (54)	15 (50)	2 (25)	8 (89)	7 (87)	5 (38)

NOS, not otherwise specified

### Overall, *Cancer*-Specific, and Other-Cause Mortality in Prostate Sarcoma Patients

**Fig. 3:**
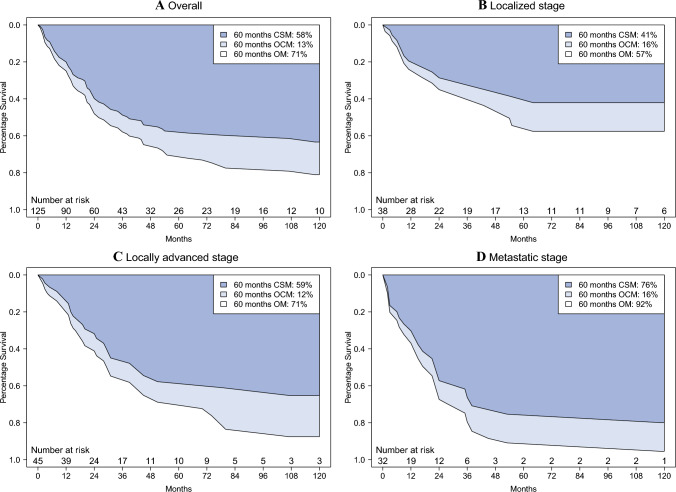
Smoothed cumulative incidence plots addressing cancer-specific mortality (CSM), other-cause mortality (OCM), and overall mortality (OM) of adult patients with sarcoma of the prostate **A** across all stages, with **B** localized, **C** locally advanced, and **D** metastatic stage. CSM, cancer-specific mortality; OCM, other-cause mortality; OM, overall mortality

In the overall population of prostate sarcoma patients, the median OS was 27 months (Fig. 3A). The 5-years CSM versus OCM rates were 58% versus 13%, which resulted in in an OM rate of 71%. Of all the deaths recorded during 5 years of follow-up evaluation, cancer-specific deaths accounted for 82% in the overall population.

### Overall, *Cancer*-Specific, and Other-Cause Mortality in Prostate Sarcoma Patients According to Stage

In stage-specific analyses, the median OS was 54 versus 30 versus 21 months in localized versus locally advanced versus metastatic stage prostate sarcoma patients, respectively (Fig. 3B–D). The 5-years CSM versus OCM rates were 41 versus 16% for a total OM rate of 57% in localized, 59 versus 12% for a total OM rate of 71% in locally advanced, and 76 versus 16% for a total OM rate of 92% in metastatic stage patients. Of all deaths recorded during 5 years of follow-up evaluation, cancer-specific deaths accounted for 72% in localized, 83% in locally advanced, and 83% in metastatic stage patients.

### Overall, *Cancer*-Specific, and Other-Cause Mortality in Prostate Sarcoma Patients According to Histologic Subtypes

**Fig. 4:**
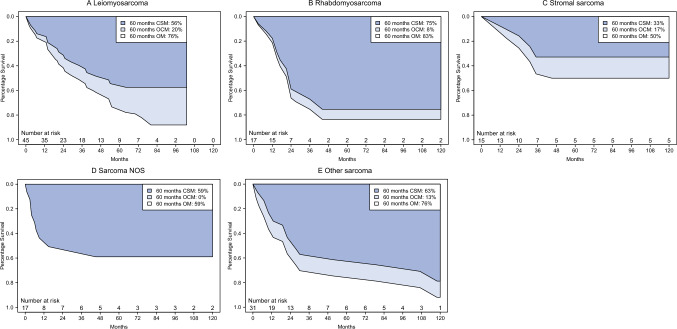
Smoothed cumulative incidence plots addressing cancer-specific mortality (CSM), other-cause mortality (OCM), and overall mortality (OM) of adult patients with **A** leiomyosarcoma, **B** rhabdomyosarcoma, **C** stromal sarcoma, **D** sarcoma not otherwise specified (NOS), and **E** other sarcoma of the prostate. CSM, cancer-specific mortality; NOS, not otherwise specified; OCM, other-cause mortality; OM, overall mortality

In histologic subtype-specific analyses, the median OS was 15 versus 21 versus 24 versus 38 versus 104 months in sarcoma NOS versus other sarcoma versus rhabdomyosarcoma versus leiomyosarcoma versus stromal sarcoma patients, respectively (Fig. 4A–E). The 5-years CSM versus OCM rates were 56 versus 20% for a total OM rate of 76% in leiomyosarcoma, 75 versus 8% for a total OM rate of 83% in rhabdomyosarcoma, 33 versus 17% for a total OM rate of 50% in stromal sarcoma, 59 versus 0% for a total OM rate of 59% in sarcoma NOS, and 63 versus 13% for a total OM rate of 76% in other sarcoma patients. Of all the deaths recorded during 5 years of follow-up evaluation, cancer-specific deaths accounted for 74% in leiomyosarcoma patients, 90% in rhabdomyosarcoma patients, 66% in stromal sarcoma patients, 100% in sarcoma NOS patients, and 83% in other sarcoma patients.

### Uni- and Multivariable Cox Regression Models Addressing Overall Mortality

In univariable Cox regression models, metastatic stage (hazard ratio [HR] 2.79; 95% confidence interval [CI] 1.54–5.08; *p* < 0.001) and unknown stage (HR 3.38; 95% CI 1.47–7.75; *p* = 0.004) were significantly associated with higher OM rates than localized stage (Table 3). Conversely, no differences were observed according to histologic subtype. In multivariable Cox regression models, patients with metastatic stage (HR 2.87; 95% CI 1.55–5.31; *p* < 0.001) and unknown stage (HR 2.94; 95% CI 2.20–7.21; *p* = 0.019) exhibited the highest OM rates. Additionally, after adjustment for stage at presentation, stromal sarcoma relative to leiomyosarcoma histology received an independent predictor status of lower OM (HR 0.41; 95% CI 0.18–0.96; *p* = 0.039), but not rhabdomyosarcoma (*p* = 0.6), sarcoma NOS (*p* = 0.7), or other sarcoma histology (*p* = 0.9).

**Table 3:** Uni- or multivariable Cox regression models addressing overall mortality (OM) of adult patients with sarcoma of the prostate

Characteristics	Univariable		Multivariable	
	HR (95% CI)	*p* value	HR (95% CI)	*p* value
Stage at presentation (Ref. Localized)				
Locally advanced	1.67 (0.96–2.92)	0.072	1.50 (0.86–2.64)	0.2
Metastatic	2.79 (1.54–5.08)	< 0.001	2.87 (1.55–5.31)	< 0.001
Unknown	3.38 (1.47–7.75)	0.004	2.94 (2.20–7.21)	0.019
Histologic subtype (Ref. Leiomyosarcoma)				
Rhabdomyosarcoma	1.03 (0.53–2.01)	0.9	0.82 (0.41–1.62)	0.6
Stromal sarcoma	0.45 (0.20–1.02)	0.056	0.41 (0.18–0.96)	0.039
Sarcoma NOS	0.79 (0.38–1.65)	0.5	0.84 (0.40–1.78)	0.7
Other sarcoma	1.22 (0.72–2.06)	0.5	0.96 (0.55–1.69)	0.9

CI confidence interval; HR hazard ratio; NOS, not otherwise specified

## Discussion

In the current analyses, we examined clinicopathologic characteristics, treatment patterns, and survival rates for adult prostate sarcoma patients. Relying on a large contemporary population-based cohort of prostate cancer patients in the United States, we made several important observations.

First, prostate sarcoma represents a rare entity.^1–3^ In the current study (SEER 2004–2020), we identified 125 adult patients with primary prostate sarcoma during a period of 17 years. This study cohort represents the most contemporary cohort of prostate sarcoma patients. The largest cohort of prostate sarcoma patients was reported by Tward et al.,^11^ who identified 295 prostate sarcoma patients during a period of 42 years (SEER 1973–2014). In their cohort, both carcinosarcoma and pediatric prostate sarcomas were included alongside other sarcomas.^11^ These differences in the study cohort selection are important because carcinosarcoma versus pure sarcoma and pediatric versus adult prostate sarcoma differ in their treated natural history.^7,9,11,15^ Moreover, the more than 40-years span of this series introduces significant heterogeneity regarding staging and treatment that may undermine the ability to interpret their observations as findings that apply to a single prostate sarcoma group. Other single- or multi-institutional studies addressing prostate sarcoma relied on substantially smaller samples, ranging from only 2 to as many as 61 prostate sarcoma patients.^4–10,12,16–18^ Consequently, prostate sarcoma patients should ideally be investigated in multi-institutional or population-based analyses when cancer control outcomes represent study end points, as was done in the current study.

Second, the three most common histologic subtype categories of adult prostate sarcoma are leiomyosarcoma (*n* = 45, 36%), rhabdomyosarcoma (*n* = 17, 14%), and sarcoma NOS (*n* = 17, 14%), in that decreasing order. These observations validate previously reported distributions of histologic prostate sarcoma subtypes among historic single-institutional case series from both the Memorial Sloan Kettering Cancer Center and the M.D. Anderson Cancer Center.^4,7^ Conversely, analyses that also included pediatric patients reported a significantly higher proportion of rhabdomyosarcoma.^5,11^

Third, we identified important differences in stage distribution according to histologic prostate sarcoma subtypes. Metastatic stage was the most common among the rhabdomyosarcoma patients (44%) and the least common among the leiomyosarcoma (21%) and stromal sarcoma (20%) patients. To the best of our knowledge, no contemporary study has addressed stage distribution according to histologic prostate sarcoma subtypes. Conversely, the historic single-institutional case series by Sexton et al.,^4^ Janet et al.,^5^ and Wang et al.^9^ indicated the highest proportion of metastatic stage among leiomyosarcoma patients. However, these case series included only 10 to 25 prostate sarcoma patients and were therefore limited in their robustness and ability to generalize their observations.

Fourth, we identified important differences in treatment patterns according to histologic subtypes. In our contemporary study cohort, most of the rhabdomyosarcoma patients received the combination of systemic and radiation therapy with or without radical surgery. Conversely, most of the leiomyosarcoma and stromal sarcoma patients underwent radical surgery with or without radiation. Indeed, multimodal treatment is preferred in prostate sarcoma management.^8,10^ However, to the best of our knowledge, no previous study reported treatment rates according to histologic prostate sarcoma subtypes. Consequently, direct comparisons of treatment patterns recorded in the current study are not possible because no previous analysis addressed this end point.

Fifth, we examined survival rates in the overall population of adult prostate sarcoma patients. Specifically, the median OS of prostate sarcoma was 27 months and the 5-years OM rate was 71%. Cancer-specific deaths accounted for 82% of all deaths recorded during 5 years of follow-up evaluation. The aforementioned observations recorded for the most contemporary population-based cohort of prostate sarcoma patients validate not only previous analyses that indicated poor survival of prostate sarcoma patients in small and historic series,^4,5,7–10,17^ but also our hypothesis that CSM rates are higher than OCM rates for prostate sarcoma patients.

Sixth, we identified important differences in survival according to disease stage at presentation. In stage-specific analyses, the median OS was 54 months for localized versus 30 months for locally advanced versus 21 months for metastatic stage prostate sarcoma patients. Even after adjustment for histologic subtype in multivariable Cox regression models, metastatic (HR 2.87) and unknown (HR 2.94) stages represented the strongest predictors of OM. Moreover, during 5 years of follow-up evaluation, cancer-specific deaths accounted for 72% in localized, 83% in locally advanced, and 83% in metastatic stage patients of all recorded deaths. It is noteworthy that among prostate sarcoma patients, the majority of deaths are attributable to prostate cancer. Invariably, OCM plays a minor role for prostate sarcoma patients.

Finally, we relied on histologic subtype-specific survival analyses. Specifically, the median OS was 15 months for sarcoma NOS versus 21 months for other sarcoma versus 24 months for rhabdomyosarcoma versus 38 months for leiomyosarcoma versus 104 for stromal sarcoma patients. Of all the deaths recorded during 5 years of follow-up evaluation, cancer-specific deaths accounted for 100% in sarcoma NOS, 90% in rhabdomyosarcoma, 83% in other sarcoma, 74% in leiomyosarcoma, and 66% in stromal sarcoma patients. After adjustment for stage at presentation, in multivariable Cox regression models, stromal sarcoma patients exhibited more favorable OM rates (HR 0.41; *p* = 0.039) than all other histologic subtype groups.

To the best of our knowledge, we are the first to compare stromal sarcoma directly with leiomyosarcoma in multivariable Cox regression models. Consequently, no direct comparison with any previous report is possible. However, survival analyses by Wang et al.^9^ indicated less favorable OS for leiomyosarcoma patients than for other prostate sarcoma patients. Although we observed no statistically significant survival differences among other pairwise comparisons according to histologic subtypes, it is important to note that potential survival differences among other histologic subtypes cannot be ruled out due to the limited number of observations and events.

Taken together, the findings show that prostate sarcoma represents an extremely rare primary cancer with a poor prognosis. Relying on the most contemporary population-based cohort of prostate sarcoma patients, the current analyses provide important and novel insights into the clinicopathologic characteristics, treatment patterns, and survival rates of prostate sarcoma patients. These observations may help clinicians caring for prostate sarcoma to better predict the treated natural history. Consequently, these results may be beneficial in clinical decision-making. Additionally, the aforementioned findings are also of great epidemiologic value because they provide the most contemporary estimate of prostate sarcoma stage distribution and cancer control outcomes.

Nevertheless, the study had limitations. First and foremost, the study relied on an observational study design. However, this limitation is shared with all previous studies that used retrospective multi-institutional registries^10^ or population-based databases, such as the SEER.^11,15,19–22^ Second, despite the large scale of the SEER database, the number of patients with a diagnosis of prostate sarcoma and its histologic subtypes, including leiomyosarcoma, rhabdomyosarcoma, stromal sarcoma, and sarcoma NOS, is limited due to the rarity of these malignancies. Third, the ICD-O-3 histology codes are extracted from patient records. No central validation of pathologic review was completed. Nevertheless, the intrinsic biases linked with this methodology are applicable to all histologic subtypes. Fourth, the amount of detail within the SEER database is limited. For example, detailed information regarding tumor grade and size as well as treatment sequence was not available for every patient. Consequently, these covariates were not included in the current analyses. Finally, the SEER database does not provide earlier cancer-control end points than OM, CSM, or OCM. Therefore, other study end points that could be equally as interesting, such as time to local recurrence or metastasis, could not be investigated within the current database.

## Conclusions

Four major histologic subtypes were identified. In most adult sarcoma patients, treatment patterns vary according to histology, from multimodal therapy to radical prostatectomy alone. These treatment differences reflect equally important heterogeneity in survival patterns.
